# Phenotypic Alterations in Hippocampal NPY- and PV-Expressing Interneurons in a Presymptomatic Transgenic Mouse Model of Alzheimer’s Disease

**DOI:** 10.3389/fnagi.2016.00327

**Published:** 2017-01-19

**Authors:** Ian Mahar, Marilia Silva Albuquerque, Siddhartha Mondragon-Rodriguez, Chelsea Cavanagh, Maria Antonietta Davoli, Jean-Guy Chabot, Sylvain Williams, Naguib Mechawar, Rémi Quirion, Slavica Krantic

**Affiliations:** ^1^Douglas Mental Health University InstituteVerdun, QC, Canada; ^2^McGill Group for Suicide Studies, Douglas Mental Health University InstituteVerdun, QC, Canada; ^3^Integrated Program in Neuroscience, McGill UniversityMontreal, QC, Canada; ^4^Laboratory of Biomedicine and Biotechnology, School of Arts, Sciences and Humanities, Universidade de São PauloSão Paulo, Brazil; ^5^Graduation Course on Pharmacology, Institute of Biomedical Sciences, Universidade de São PauloSão Paulo, Brazil; ^6^Research Group on Neuropharmacology of AgingSão Paulo, Brazil; ^7^CONACYT National Council for Science and TechnologyMéxico city, Mexico; ^8^UNAM Developmental Neurobiology and Neurophysiology, Institute of Neurobiology, National Autonomous University of MéxicoQuerétaro, Mexico; ^9^Centre de Recherche des Cordeliers, Sorbonne Universités, UPMC Univ Paris 06, INSERM, Université Paris Descartes, Sorbonne Paris Cité, UMR_S 1138Paris, France

**Keywords:** Alzheimer’s disease, neuropeptide Y, parvalbumin, hippocampal sub-regions, pre-plaque

## Abstract

Interneurons, key regulators of hippocampal neuronal network excitability and synchronization, are lost in advanced stages of Alzheimer’s disease (AD). Given that network changes occur at early (presymptomatic) stages, we explored whether alterations of interneurons also occur before amyloid-beta (Aβ) accumulation. Numbers of neuropeptide Y (NPY) and parvalbumin (PV) immunoreactive (IR) cells were decreased in the hippocampus of 1 month-old TgCRND8 mouse AD model in a sub-regionally specific manner. The most prominent change observed was a decrease in the number of PV-IR cells that selectively affected CA1/2 and subiculum, with the pyramidal layer (PY) of CA1/2 accounting almost entirely for the reduction in number of hippocampal PV-IR cells. As PV neurons were decreased selectively in CA1/2 and subiculum, and given that they are critically involved in the control of hippocampal theta oscillations, we then assessed intrinsic theta oscillations in these regions after a 4-aminopyridine (4AP) challenge. This revealed increased theta power and population bursts in TgCRND8 mice compared to non-transgenic (nTg) controls, suggesting a hyperexcitability network state. Taken together, our results identify for the first time AD-related alterations in hippocampal interneuron function as early as at 1 month of age. These early functional alterations occurring before amyloid deposition may contribute to cognitive dysfunction in AD.

## Introduction

Alzheimer’s disease (AD) is an age-related neurodegenerative disorder characterized by progressive loss of cognitive and executive functions. AD develops over decades, and although overt stages are commonly studied, little is known about the mechanisms occurring in the earliest stages (Prince et al., 2014). Understanding these upstream mechanisms is crucial for identifying early diagnostic biomarkers, as well as therapeutic targets that could help modify disease progression more efficiently.

The main histological hallmarks of AD are deposition of amyloid beta (Aβ) peptides into extracellular amyloid plaques and intracellular accumulation of hyperphosphorylated tau protein (neurofibrillary tangles) (Hardy and Selkoe, 2002; Hardy, 2006). The prevailing theory regarding the cause of AD is the amyloid cascade hypothesis, which posits that overproduction of Aβ from amyloid precursor protein (APP) initiates a series of events, including synaptic dysfunction, microglial and astrocytic activation and hyperphosphorylation of tau, which culminates in widespread neuronal death and neurodegeneration (Hardy and Selkoe, 2002). In addition to the production of Aβ, processing of APP along the amyloidogenic pathway liberates beta C-terminal fragment (βCTF), the rate-limiting precursor to Aβ (Chow et al., 2010). βCTF is also known to have neurotoxic properties independent of the Aβ region (Lee et al., 2000) and was associated with cortical atrophy when cells expressing βCTF were transplanted in the brains of newborn mice (Neve et al., 1992). Furthermore, transgenic mice overexpressing βCTF also display extensive hippocampal neuronal degeneration (Kammesheidt et al., 1992; Oster-Granite et al., 1996), suggesting that Aβ may not be solely responsible for AD pathology. Because βCTF accumulates prior to Aβ (Cavanagh et al., 2013) and because its deleterious effects such as neurodegeneration (Kammesheidt et al., 1992) and synaptic abnormalities (Oster-Granite et al., 1996) resemble those associated with Aβ, AD-related pathological alterations may arise even earlier (i.e., before Aβ accumulation) than initially thought.

It has been recently proposed that neurodegeneration may stem from neuronal hyperexcitability. The regulatory mechanisms controlling the excitability state of hippocampal neuronal networks and preventing a transition towards a state of excitability (i.e., a network hyperexcitability state) have been examined in the hippocampus, yet the underlying molecular and biochemical mechanisms remain unknown. This functional regulation operates under physiological conditions but is lost under pathological conditions (Niedringhaus et al., 2015). Hyperexcitability, as manifested by epileptiform activity, is observed at the earliest stages of AD in both humans and mice (Palop et al., 2007; Gleichmann et al., 2011). Based on this evidence, it has been hypothesized that neuronal death is a consequence, rather than cause, of epileptic seizures (Palop et al., 2007). Further, the Aβ-mediated increase in hippocampal network excitability is associated with a widespread increase in excitatory activity, with subsequent negative impact on learning and memory (Palop et al., 2007; Palop and Mucke, 2009). A similar increase in excitability was detected after *in vivo* treatment of 1.5–2 month-old TgCRND8 mice with pentylenetetrazole, a drug that inhibits type-A γ-aminobutyric acid (GABA) receptors (Del Vecchio et al., 2004). This may be due to the AD-related decrease in parvalbumin (PV) neuron activation, which leads to hyperexcitability (Verret et al., 2012). More recently, using the same AD model, we have reported that subtle alterations in synchronization of intrinsic hippocampal gamma and theta oscillations are detected as early as 1 month of age in TgCRND8 mice (Goutagny et al., 2013). Given that hippocampal network activity is coordinated by GABAergic neurons (Cobb et al., 1995; Lawrence and McBain, 2003; Mann and Paulsen, 2007; Amilhon et al., 2015), GABAergic neuronal dysfunction may lead to network over-excitation, and thus underlie an increased susceptibility to seizures. Similarly, soluble Aβ can disrupt excitatory-inhibitory balance, a specific AD-linked (apoE4) genotype has been associated with impairment of GABAergic interneurons, and GABAergic activation-induced hyperpolarization prevents Aβ-related toxicity (Huang and Mucke, 2012; Paula-Lima et al., 2013; Nava-Mesa et al., 2014). However, the dysfunction of GABAergic interneurons in the earliest AD-associated state of hippocampal excitability and network synchronization has not been assessed.

Here, we investigated putative GABAergic interneuronal impairment in 1 month-old TgCRND8 mice. Aβ pathology progresses during the course of aging, and 6 month-old TgCRND8 mice have high levels of Aβ and severe plaque load in many brain regions, including the hippocampus (Chishti et al., 2001). Although Aβ plaques are undetectable before 3 months of age (Chishti et al., 2001), βCTF, the first cleavage product of APP is expressed already at 1 month (Cavanagh et al., 2013; Goutagny et al., 2013). Impaired performance in cognitive tasks is first detectable at 2 months of age, but only when more sensitive tests such as object recognition tasks are used, as 2 month-old TgCRND8 mice are unimpaired on a Morris water maze task (Francis et al., 2012). We chose to examine TgCRND8 mice at 1 month of age because it corresponds to the AD pathogenesis stage at which alterations in hippocampal neuronal activity may be first detectable, as no difference has been detected between 15 days-old TgCRND8 mice and control littermates (Goutagny et al., 2013). We focused on neuropeptide Y (NPY; Palop et al., 2011) and PV (Verret et al., 2012) hippocampal interneurons, as both subtypes have been shown to be particularly affected in AD. Moreover, NPY and PV interneurons are critically involved in the control of hippocampal excitability (Palop et al., 2007) and network synchronization (Verret et al., 2012; Amilhon et al., 2015), respectively.

## Materials and Methods

### Animals

All experiments followed the policies and guidelines of the Canadian Council on Animal Care and the animal care regulations of McGill University. TgCRND8 mice bear Swedish KM670/671NL and Indiana V717F mutations in the hAβAPP-encoding gene and overexpress human Aβ by 3–4 months. Male TgCRND8 and non-transgenic (nTg) mice were maintained on an outbred C3H/C57BL6 background and kept on a 12 h light/dark cycle with food and water *ad libitum*.

### βCTF ELISA

βCTF content was quantified from hippocampal homogenates from Tg mice, as preliminary examination indicated that it is undetectable in nTg mice (data not shown). This was expected, as the antibody used in the enzyme-linked immunoabsorbent assay (ELISA) kit (IBL International, Japan) is directed against the human βCTF protein. Hippocampi from male TgCRND8 mice (*n* = 8 mice) were dissected on ice, snap frozen and stored at −80°C until proteins were extracted. Hippocampus samples were homogenized in radioimmunoprecipitation assay (RIPA) buffer (100 μl per hippocampus) and left on ice for 30 min. The samples were then centrifuged for 10 min at 1000 g at 4°C. The supernatants were collected and protein concentrations were determined using a BCA assay kit (Pierce, Rockford, IL, USA). The βCTF content was then assessed in triplicates using an ELISA kit (IBL International, Japan) as per manufacturer’s instructions. Briefly, samples were diluted in enzyme immunoassay (EIA) buffer (1:100) and standards were prepared as directed. 100 μl of each sample was loaded into the appropriate wells and incubated overnight at 4°C. The plate was then washed seven times using diluted wash buffer. One hundred microliter of labeled antibody solution was loaded into each well, and the plate was incubated for 1 h at 4°C. The plate was subsequently washed nine times and 100 μl of chromogen was added to each well. After 30 min of incubation in the dark at room temperature, 100 μl of stop solution was added to each well, and the plate was analyzed using a plate reader at 450 nm against the reagent blank.

### Immunohistochemistry

Additional male mice (*n* = 5 mice per experimental group) were anesthetized by pentobarbital and transcardially perfused (PBS followed by 4% PFA). Brains were stored in fixative for 24 h at 4°C, then in a sucrose solution (30% in PBS) for 3 days at 4°C, frozen using dimethylbutane and stored at −80°C. Brains were sliced coronally using a freezing microtome at 40 μm for light microscopy, or using a vibrating microtome at 50 μm for fluorescence microscopy, and free-floating sections were stored in a cryopreservative solution (3:3:4 glycerol:ethylene glycol:PBS) at −20°C in preparation for IHC (IHC) staining. The serial sectioning fraction for IHC was 1/8). Washes in PBS preceded all steps except primary antibody addition. All steps were at room temperature unless otherwise specified.

For immunofluorescence, sections were incubated for 5 min with formic acid (90%). They were then incubated for 1 h with PBS containing 1% normal goat serum, 0.25% Triton X-100 and 0.45% gelatin. Sections were further incubated overnight at 4°C with either FCA3340 (Barelli et al., 1997) to detect Aβ (Millipore 171608) or CT20 (Millipore 171610) to detect βCTF (rabbit, 1:1000 dilution for both). Sections were incubated for 2 h with secondary antibody (Alexa 555-conjugated goat anti-rabbit IgG; ThermoFisher A-21428, 1:2000). Sections were incubated with DAPI (1:10,000 dilution) for 15 min at room temperature. Sections were then mounted on glass slides with Fluoromount G. Fluorescence was visualized using an epifluorescence (Axioplan2, Zeiss) microscope.

For the study of neuronal populations, sections were incubated in PBS with 0.2% Triton X-100 for 1.5 h, followed by 3% H_2_O_2_ prepared in PBS for 10 min. The sections were blocked with 2% normal serum in PBS + 0.2% Triton X-100 for 1 h. Primary antibodies (NeuN: mouse, 1:200, Millipore MAB377; NPY: rabbit, 1:2000, Abcam ab10980; PV: rabbit, 1:5000, Abcam ab11427) were applied overnight at 4°C, followed by biotinylated secondary antibodies (Vector BA-1000, 1:200; BA-2001, 1:500) for 1 h. The immunolabeled product was visualized using the avidin-biotin complex method (Vectastain elite ABC Kit, Vector Laboratories; 30 min), followed by DAB (for NeuN) or SG (for NPY and PV) chromogen development (Vector Laboratories). Stained sections were mounted on slides, dried overnight, dehydrated in ethanol, cleared in xylene and coverslipped. Parallel sections from the same animals were used across immunohistochemical experiments.

### Cell Quantifications

Immunoreactive (IR) cell somata were counted in each hippocampal region and sub-region (from Bregma −1.06 mm to Bregma −3.88 mm; Franklin and Paxinos, 2007), on a Nikon Eclipse E600 (Kanagawa, Japan) microscope with a 20X objective, by an experimenter blind to group identity. Anatomical regions were determined as per Franklin and Paxinos (2007). CA1 and CA2 were combined (CA1/2) due to the relatively amorphous boundary separating these two regions. Intra-subject counts by an additional experimenter (on a Leica DM 2500 microscope) to confirm count/total accuracy correlated significantly (*p* = 0.0062), with Pearson r and intraclass correlation values >0.5. The serial section fraction was 1/8. Cell quantifications are shown as numbers of cells per section to correct for number of sections and for consistency between experiments. Pairwise comparisons between nTg and TgCRND8 animals in hippocampal regions and sub-regions were performed using unpaired *t*-tests, with Welch’s correction applied when required.

For GABAergic markers, we were able to count each labeled cell in order to quantify. However, given the large number of NeuN-labeled cells, it was necessary to use stereological estimates to quantify this marker, as in previous studies (Pham et al., 2003). Although this method relies on estimates as opposed to absolute cell counts, and is not appropriate for quantification of all hippocampal cell types (Noori and Fornal, 2011), it is an optimal quantification method for this larger cellular population. NeuN-IR quantifications were performed by an experimenter blind to group identity on an Olympus BX51 microscope with a motorized stage, using StereoInvestigator software (MBF Bioscience). The stereological parameters (optimized for the NeuN-IR population) were: sampling grid area 28440 μm^2^, counting frame 25 μm × 25 μm, dissector height 10 μm, guard zone 1 μm. Volume was assessed using a Cavalieri estimator (20 μm grid spacing) corrected for overprojection. Average coeffecients of error for Cavalieri probes were 0.025 for CA1/2 and CA3 and 0.033 for dentate gyrus (DG), and for optical fractionator (Gundersson *m* = 1) were 0.051 for CA1/2, 0.060 for CA3 and 0.054 for DG.

### Electrophysiology

Tg and nTg mice (aged 30–35 days; *n* = 6/group) were decapitated, and the brain was rapidly removed and placed in ice-cold high sucrose artificial CSF (ACSF) solution (in mM: 252 sucrose, 3 KCl, 2 MgSO_4_ 24 NaHCO_3_, 1.25 NaH_2_PO_4_, 1.2 CaCl_2_ and 10 glucose) and bubbled with carbogen (95% O_2_ and 5% CO_2_). The cerebellum and frontal cortex were removed with a razor blade, and the hemispheres were separated and allowed to recover for 2–3 min in the oxygenated sucrose solution. Complete septo-hippocampal isolate was then removed from the remaining hemisection as described previously (Goutagny et al., 2009). After dissection, the complete septo-hippocampal preparation was left at room temperature in ACSF bubbled with carbogen for 60 min. For recording, the preparation was transferred quickly to a custom-made submerged recording chamber. Recordings were performed at 30–32°C after an additional 30 min period in the chamber. The preparation was continuously perfused with ACSF (25 ml/min, in mM: 126 NaCl, 24 NaHCO_3_, 10 glucose, 4.5 KCl, 2 MgSO_4_, 1.25 NaH_2_PO_4_ and 2 CaCl_2_, pH 7.4, with 95% O_2_/5% CO_2_) via a gravity-fed perfusion system and maintained at 30–32°C. Local field potentials were recorded using glass micropipettes (2–6 MΩ) filled with ACSF. Signals were recorded through a differential AC amplifier (A-M Systems), filtered online (0.1–500 Hz), and sampled at 5 KHz. All drugs came from aliquots of stock solutions (stored at −80°C) and were added to the perfusing artificial ACSF at the concentrations indicated. Base line recording lasted for 20 min followed by 100 s of pharmacological stimulation (4AP at 150 μM) and 20 min recovery after stimulation. Changes in theta power were measured in mV^2^/Hz. Pairwise comparisons were performed with *t*-tests. For all experiments, a *p* value of ≤0.05 considered statistically significant. Bar graphs show experimental mean, with error bars indicating standard error of the mean.

## Results

### Immunohistochemical Assessment of Aβ and βCTF in the Hippocampus of 1 Month-Old TgCRND8 Mice

**Figure 1:**
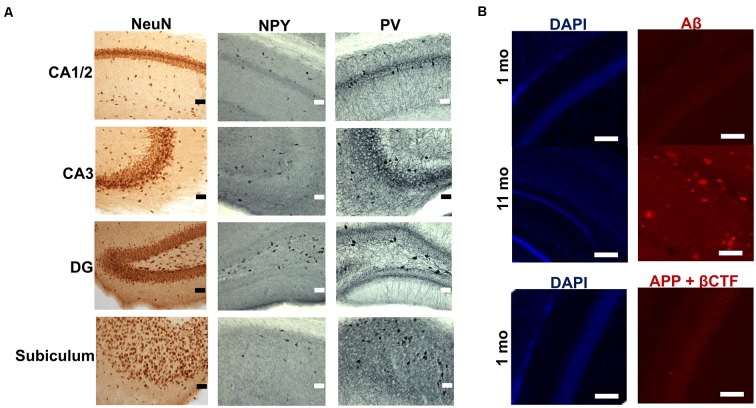
**Expression of neuronal markers and amyloid-beta precursor protein (APP) cleavage products in the hippocampus of 1 month-old TgCRND8 mice. (A)** Micrographs of immunohistochemical labeling of hippocampal neurons for NeuN, neuropeptide Y (NPY) and parvalbumin (PV) in CA1 and 2, CA3, dentate gyrus (DG) and subiculum (SUB) hippocampal regions. Scale bar = 50 μm. **(B)** Amyloid-beta (Aβ) and C-terminal fragment/Amyloid precursor protein intracellular domain (CTF/AICD) expression as assessed by FCA3340 and CT20 antibodies, respectively. Anatomically matched hippocampal sections of CA1/2 sub-region of 11 month-old TgCRND8 mice were used as a positive control. Scale bar = 100 μm.

Immunohistochemical staining for three neuronal markers (NeuN, NPY and PV) revealed distributional patterns in CA1/2, CA3, DG and subiculum (Figure 1A) that have been described previously (Albuquerque et al., 2015). Assessment of Aβ and amyloid-plaques using the FCA3340 antibody, which specifically recognizes human Aβ but not APP (Barelli et al., 1997), indicated no immunolabeling in hippocampus sections obtained from 1 month-old TgCRND8 mice, in contrast to the abundance of plaques present in the hippocampus of 11 month-old TgCRND8 mice, used as a positive control (Chishti et al., 2001; Figure 1B). However, the CT20 antibody which recognizes both human and murine full length APP and its C-terminal fragments (CTF) α, β and amyloid precursor protein intracellular domain (AICD) but not Aβ, revealed IR cells, notably in the pyramidal cell layer of the CA1/CA2 region (Figure 1B). CT20-IR cells displayed punctate cytoplasmic labeling (Figure 1B). Combined, the labeling data obtained with FCA3340 and CT20 indicated that immunoreactivity likely arises from the presence of APP and its first cleavage products (CTFs) in the absence of Aβ. Since the observed putative CTF-IR could arise from different fragments (αCTF, βCTF or AICD) generated along either the amyloidogenic and non-amyloidogenic pathways, we performed a selective βCTF ELISA to assess whether the CT20-IR could be due specifically to the βCTF. The presence of βCTF was confirmed and quantified (1.47 pg βCTF/mg protein ± 0.22, range 0.80–2.43 pg βCTF/mg protein) by ELISA in the hippocampal tissue of TgCRND8 mice aged 1 month.

### Global Phenotypic Analysis of the Composition of Selected Hippocampal Neuronal Populations

**Figure 2:**
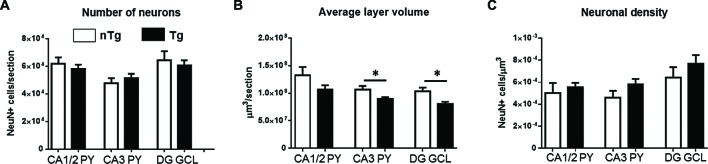
**Quantification of NeuN-immunoreactive neurons in the hippocampus of 1 month-old TgCRND8 mice.** Number of neurons **(A)**, average layer volume **(B)** and neuronal density **(C)** in the studied hippocampal sub-regions (CA1/2, CA3 and DG) were examined. nTg, non-transgenic; Tg, transgenic. *p < 0.05.

Phenotypic analysis along the rostro-caudal axis (−0.94 to −2.86 mm from Bregma) using the neuronal marker NeuN indicated that both total number (Figure 2A) and density (Figure 2C) of neurons were similar between genotypes in all studied regions of the hippocampus (CA1/2, CA3 and DG). Interestingly, structural volume was significantly decreased for TgCRND8 mice in CA3 (*p* = 0.041) and DG (*p* = 0.017) but not CA1/2, as compared to controls (Figure 2B).

**Figure 3:**
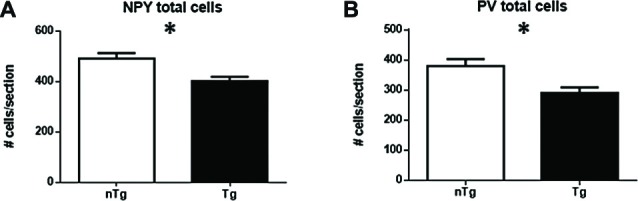
**Quantification of NPY- (A)**, and PV-immunoreactive (IR; **B**) cells in the overall hippocampus for nTg and Tg animals. *p < 0.05.

However, when assessing specifically NPY (Figure 3A; *p* = 0.016) or PV (Figure 3B; *p* = 0.016) subpopulations, a significant decrease in numbers of IR-cells was found in TgCRND8 mice compared to age-matched nTg littermates.

### Regional and Sub-Regional Distribution of NPY- and PV-Expressing Neurons

**Figure 4:**
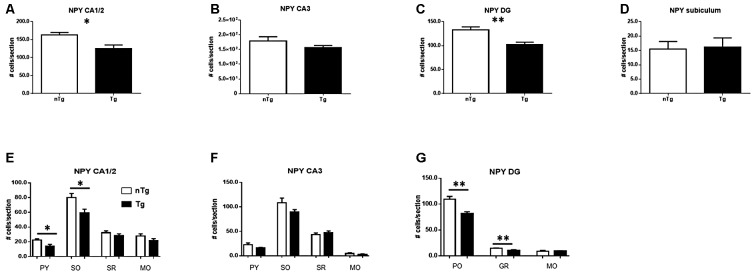
**Quantification of NPY-IR cells in CA1/2 (A)**, CA3 **(B)**, dentate gyrus (DG; **C**) and subiculum **(D)**, as well as sub-regions within CA1/2 **(E)**, CA3 **(F)** and DG **(G)**. nTg, non-transgenic; PO, polymorphic layer; SO, stratum oriens; SR, stratum radiatum; Tg, transgenic. *p < 0.05; **p < 0.01.

A more detailed analysis indicated that the overall number of NPY neurons is decreased in hippocampal sub-regions CA1/2 (*p* = 0.021) and DG (*p* = 0.0033), but not CA3 or subiculum (Figures 4A–D) of TgCRND8 mice as compared to controls. The most affected sub-regions in CA1/2 were the stratum pyramidale (*p* = 0.021) and oriens (*p* = 0.026) layers (Figure 4E). No significant difference was seen between genotypes in the layers of CA3 (Figure 4F). By contrast, in the DG, the polymorphic layer (PO; *p* = 0.0031) and granule cell layer (GR; *p* = 0.0051) showed alterations in TgCRND8 mice, whereas the molecular layer (MO) was unaffected (Figure 4G).

**Figure 5:**
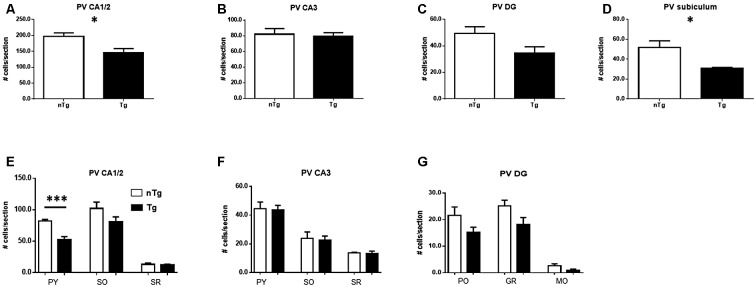
**Quantification of PV-IR cells in CA1/2 (A)**, CA3 **(B)**, dentate gyrus (DG; **C**) and subiculum **(D)**, as well as sub-regions within CA1/2 **(E)**, CA3 **(F)** and DG **(G)**. nTg, non-transgenic; PO, polymorphic layer; SO, stratum oriens; SR, stratum radiatum; Tg, transgenic. *p < 0.05; ***p < 0.001.

An analogous analysis of PV-expressing neuronal sub-populations showed a significant decrease in the number of these neurons in CA1/2 (Figure 5A; *p* = 0.017) and subiculum (Figure 5D; *p* = 0.030) whereas CA3 (Figure 5B) and DG (Figure 5C) were not significantly affected. Among the layers analyzed in these sub-regions (Figures 5E–G), the number of PV-expressing neurons in TgCRND8 mice was significantly decreased only in the pyramidal cell layer of the CA1/2 region (Figure 5E; *p* = 0.0004).

A recent study reported that PV-expressing neurons at the interface between CA1/2 and subiculum are the primary regulators of hippocampal neuronal network oscillations at theta frequencies (Amilhon et al., 2015). Therefore, we decided to focus further on the PV-expressing sub-population of interneurons.

### Assessment of Amyloid in PV-Expressing Neurons

**Figure 6:**
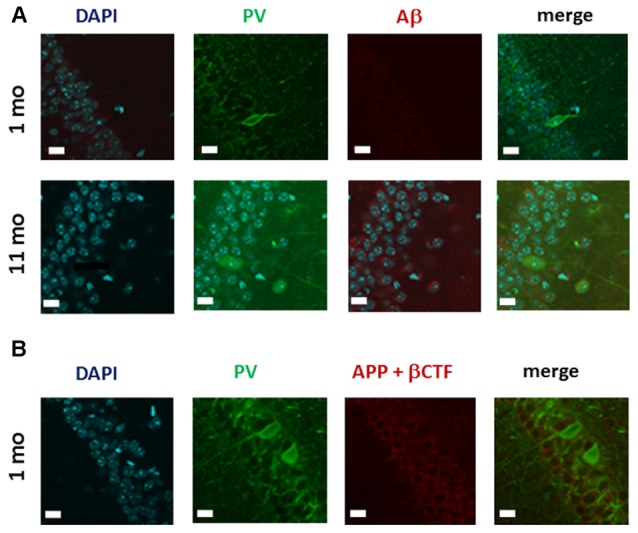
**Analysis of the co-expression of APP cleavage products and PV-IR in CA1 hippocampal neurons of 1 month-old TgCRND8 mice. (A)** Micrographs of immunohistochemical labeling of hippocampal neurons for PV and Aβ; anatomically matched sections from 11 month-old mice were used as a positive control. Note that in contrast to the absence of Aβ-IR in 1 month-old TgCRND8 mice, intracytoplasmic Aβ is clearly detectable in the pyramidal layer (PY) of the CA1 region of 11 month-old TgCRND8 mice. **(B)** Analysis of Aβ/CTF expression in CA1 hippocampal PV neurons in 1 month-old TgCRND8 mice. As Aβ was undetectable when assessed with the selective FCA3340 Aβ antibody, and enzyme-linked immunoabsorbent assay (ELISA) detected a substantial amount of βCTF, this immunoreactivity reveals likely the expression of βCTF. However, no PV-IR neurons co-expressing βCTF were observed. Scale bar = 10 μm.

To explore the mechanisms of the observed decrease in the number of PV-expressing interneurons in 1 month-old TgCRND8 mice, we used a double-labeling approach to determine whether, in the absence of Aβ (Figure 1B), PV-expressing neurons may co-express βCTF. Using anti-PV and FCA3340 antibodies, we first confirmed the absence of Aβ in the PV-IR neurons located in the pyramidal layer (PY) of CA1 in 1 month-old TgCRND8 mice (Figure 6A). By contrast, in the anatomically matched hippocampal region from 11 month-old TgCRND8 mice, which was used as a positive control, cytoplasmic Aβ labeling was revealed in pyramidal neurons.

As our ELISA data demonstrated βCTF expression in the hippocampus in 1 month-old TgCRND8 mice, we next asked whether PV-expressing neurons may also express βCTF. Our double-labeling approach indicated that CTF-IR (as revealed by the CT20 antibody) does not co-localize with PV-IR (Figure 6B). Interestingly, in 1 month-old TgCRND8 mice, APP and CTFs were apparently localized in the upper portion of the pyramidal cell layer where PV-IR neurons were virtually never detected (Figure 6B). Indeed, PV-expressing neurons were localized in the lower portion of the PY (Figure 6B).

### Functional Assessment of Hippocampal Network Excitability

Transgenic mouse models of AD at 5 months of age are characterized by high levels of Aβ peptides potentially leading to network hyperexcitability (Palop et al., 2007). However, our recent data examining Tg mice at 1 month of age has not indicated the presence of Aβ peptide (Goutagny et al., 2013); instead, we observed elevated βCTF and decreased numbers of hippocampal PV-expressing neurons in the current study. Aiming to assess whether these alterations could account for network hyperexcitability (changes in theta amplitude, theta frequency and burst (seizure) events), we utilized 4-aminopyridine (4AP) in a complete septo-hippocampal preparation (Goutagny et al., 2009). The hyperexcitability network state is achieved by blocking primarily K_v_1 channels, which consequently induces activity reflecting the firing of GABA-releasing cells and is sustained by GABA_A_ receptor signaling (Avoli and de Curtis, 2011). Our hypothesis was that 4AP would reveal a state of hyperexcitability in juvenile TgCRND8 mice.

**Figure 7:**
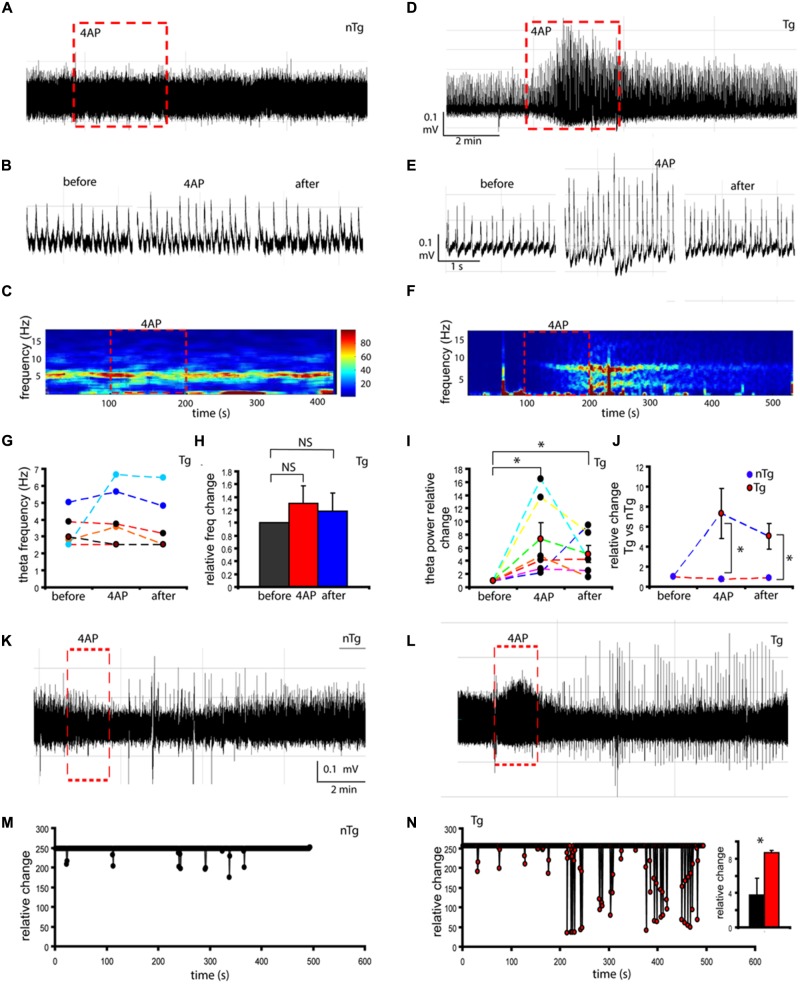
**Electrophysiological monitoring of CA1/subiculum neurons in response to 4AP challenge.** Raw traces of theta oscillations recorded in CA1/subiculum area using septo-hippocampal preparations from non-transgenic (nTg; **A**) and transgenic (Tg) mice **(D)**. In nTg mice, 150 μM-4AP treatments did not change theta amplitude (**A**, red square). Conversely, in TgCRND8 mice 4AP treatments altered theta amplitude (**D**, red square). Magnification of raw theta activity before, during and after 4AP treatment in nTg **(B)** and TgCRND8 mice **(E)**. Power spectrum analysis of theta power and frequency in nTg **(C)** and TgCRND8 mice **(F)**. Theta frequency did not show statistically significant changes in TgCRND8 4AP-treated animals (**G,H**, different colors correspond to individual TgCRND8 samples). TgCRND8 mice reveal theta power increase during and after 4AP treatment (**I,J**, red circles denote averages, black circles denote individual TgCRND8 traces). Under 4AP challenge, elevated number of burst events was observed in TgCRND8 mice **(L)** compared to nTg **(K)**. Relative change of burst events is higher in TgCRND8 mice (**N**, representative trace of burst events after 4AP in **L**) compared to nTg mice (**M**, representative trace of burst events after 4AP in **K**). Burst events were significantly increased for TgCRND8 mice (see inset in **N**). *p < 0.05.

Treatment with 4AP did not elicit a visible change in the amplitude of theta activity in nTg mice (Figures 7A,B, red square). Conversely, in Tg mice 4AP treatment affected amplitude of hippocampal theta activity (Figure 7D, red square). Magnification of raw activity traces confirmed an increase in theta amplitude of Tg mice during 4AP treatment (Figure 7E). As shown in the spectrogram analysis, theta oscillations frequency remains stable (4.10 ± 1.68 Hz) in nTg mice when treated with 4AP (Figure 7C, red square). In contrast, although not statistically significant (*p* = 0.29), Tg mice showed changes in frequency during 4AP stimulation (Figure 7F, red square). Statistical analysis further confirmed our findings, as no significant changes were detected in frequency either during (*p* = 0.29) or after 4AP stimulation (*p* = 0.53; Figures 7G,H). Concomitantly, statistical analysis further confirmed the 7.5 ± 2.44-fold increase in theta power during 4AP stimulation in Tg mice (Figure 7I, *p* = 0.02). The increase was also significant after 4AP stimulation (Figure 7I, 5.03 ± 1.29 *p* = 0.01). The changes in theta peak power were also significant when Tg mice were compared to nTg during 4AP (Figure 7J, *p* = 0.038) and after stimulation (Figure 7J, *p* = 0.023).

At this point, the data suggested that the network state in TgCRND8 mice is closer to a hyperexcitability-like state when compared to controls. To further explore the excitability network state, we analyzed the number of burst events present in the nTg (Figure 7K) and Tg mice (Figure 7L). Our results showed an elevated number of burst events in Tg mice when treated with 4AP (Figure 7L), further supporting a hyperexcitability-like network state. In contrast, control mice showed a more stable network state (Figure 7K). Further statistical analysis confirmed a two-fold increase (2.4 ± 0.19-fold, *p* = 0.012) in relative burst events in Tg mice when compared to nTg mice (Figures 7M,N).

## Discussion

Alterations in hippocampal neuronal excitability (Del Vecchio et al., 2004; Palop and Mucke, 2010; Verret et al., 2012) and synchronization of hippocampal oscillatory activity (Goutagny et al., 2013) occur at the first stages of AD pathogenesis, via as yet poorly understood mechanisms. Here, we found that a significant decrease in the number of hippocampal NPY- and PV-IR cells in 1 month-old TgCRND8 mice coincides with the early impairment of neuronal network activity. This decrease was distributed between hippocampal regions CA1/2 and DG for NPY. The decreased number of PV-IR cells selectively affected CA1/2 and subiculum, with the PY of CA1/2 accounting almost entirely for the reduction in number of hippocampal PV-IR cells. This decrease in NPY-and PV-IR cells was observed in the absence of change in total numbers of hippocampal neurons (NeuN-IR) in the studied principal layers, suggesting that decreases in numbers of NPY- and PV-IR cells are not the result of neuronal death occurring in the studied hippocampal layers but may be related to loss of expression of particular GABAergic markers (see “Discussion” Section in Uchida et al., 2014), although contribution by specific GABAergic population cell death cannot be conclusively ruled out by the current data. We observed decreased volume of CA3 and DG, potentially representing changes in neuropil. Moreover, the change in GABAergic cells was observed at a stage wherein βCTF, the first cleavage product of APP and direct precursor of Aβ, was abundant. Our work (Goutagny et al., 2013; present study) is the first reporting on AD-related hippocampal dysfunction at such an early stage of pathogenesis. However, cortical hyperexcitability was recently correlated with βCTF (Xu et al., 2015) in 2–4 month-old Tg2576 mice prior to plaque formation (Duffy et al., 2015), thus further pointing to dysregulation of excitability as a general feature of initial AD stages. As a corollary, currently available AD mouse models should be studied at much earlier ages than have been previously examined, specifically in the context of putative early impairments of GABAergic interneurons.

Electrophysiological monitoring of theta oscillations in the distal CA1/subiculum of TgCRND8 mice revealed increased power, without change in frequency of theta oscillation in comparison to nTg littermates. These findings suggest altered control of hippocampal network excitability state as a potential mechanism of network dysfunction seen during early stages of AD development at the same age (1 month-old) and the same strain (TgCRND8) of AD mouse model (Goutagny et al., 2013). Furthermore, although 4AP triggered burst activity in neuronal populations of both genotypes, the relative change of burst events was significantly higher in TgCRND8 mice than in controls, suggesting an AD-associated hyperexcitability. Therefore, treatment with 4AP appeared to induce a prodromal network hyperexcitability-like state in the studied 1 month-old TgCRND8 mice, as previously seen in older mice of the same strain (Jolas et al., 2002; Del Vecchio et al., 2004).

Given the recently demonstrated pivotal role of PV-expressing GABAergic neurons in the generation of theta oscillations in the isolated hippocampus (Amilhon et al., 2015), these data suggest that the loss of PV-expression in GABAergic neurons may be causal to their functional impairment. Thus, the decrease in number of functional PV neurons in the absence of their death (as attested by the absence of difference in the number of hippocampal NeuN-IR neurons between genotypes) may contribute to the loss of inhibitory tone and subsequent increased excitability of pyramidal cells. As PV neurons are key for the synchronization of pyramidal cells, this loss of PV expression can likely have a critical impact on CA1 network activity. Furthermore, the observed AD-related hyperexcitability may in turn be related to the previously reported uncoupling of theta/gamma oscillations (Goutagny et al., 2013). In addition to effects on excitability, the loss of PV neurons may also influence hippocampal plasticity. Indeed, in a recent study LTP was enhanced in rat hippocampal slices after application of an α_7_–nicotinic acetylcholine receptor agonist in part through an enhancement of a GABAergic receptor subtype (Townsend et al., 2016). The effect of phenotypic changes in GABAergic neurons on synaptic plasticity should also be explored, especially given the implication of PV cells in AD (Verret et al., 2012). Notably, synaptic hyperexcitability in CA1, which has been reported in 5 month-old TgCRND8 mice (Jolas et al., 2002), is present much earlier, as is shown here at the age of 1 month, before Aβ overproduction. At this time point βCTF is detectable, and given that this protein has been previously associated with impairments in synaptic function (Nalbantoglu et al., 1997; Tamayev et al., 2012), the presence of βCTF raises the possibility that this neurotoxic protein may be affecting GABAergic subpopulations. In this light, decreased numbers of NPY-IR neurons in the stratum oriens (SO) and pyramidale of the CA1 region, combined with the significant loss of PV-IR in the PY of CA1, would likely have major consequences in terms of control of pyramidal cell excitability. Indeed, cells in the SO project to distal dendrites of CA1 pyramidal cells and provide dendritic inhibition (Maccaferri and Lacaille, 2003). It has to be stressed however that, because of the very young (1 month) age at which we assessed the putative changes in the hippocampus of TgCRND8 mice, the possibility remains that the observed decreased numbers of the PV neurons may be related to the incomplete maturation of the GABAergic neurons, rather than to their AD-related decrease. This possibility is nevertheless very unlikely, as our previous study demonstrated that at the age of 2 weeks, there is no difference in the hippocampal network activity between TgCRND8 mice and their age-matched nTg controls (Goutagny et al., 2013).

A previous phenotypic analysis of hippocampal GABAergic neuronal populations at the overt stages of AD pointed to selective loss in number or function of specific sub-types, notably neurons expressing NPY (Ramos et al., 2006; Albuquerque et al., 2015), and PV (Verret et al., 2012; Albuquerque et al., 2015). As with the aforementioned CA1 synaptic hyperexcitability, our results indicate that the change in the composition of certain GABAergic sub-populations may occur much earlier than previously believed. This is particularly striking for NPY-expressing GABAergic neurons that, by the age of 1 month (in the present study), have already decreased substantially in number, similar to 6 month-old TgCRND8 mice (Albuquerque et al., 2015) in all studied hippocampal regions. By contrast, the alteration of PV-expressing neurons appears more complex because the decreased expression of PV found in CA1 and subiculum at 1 month of age is not detectable in 6 month-old animals (Albuquerque et al., 2015). Additionally, in another AD mouse model (APP/PS1), a significant loss of PV neurons was shown in CA1/2 region of 10 month-old mice (Takahashi et al., 2010). A putative loss of PV neurons in older TgCRND8 mice cannot be completely excluded as these mice have not been studied at ages older than 6 months (Albuquerque et al., 2015).

Collectively, our data indicate that before the loss of GABAergic neurons in AD mouse models (Krantic et al., 2012; Albuquerque et al., 2015), GABAergic neurons undergo substantial alterations that potentially increase seizure susceptibility (Palop et al., 2007). These include changes in neurochemical phenotype and composition of GABAergic subpopulations, compatible with the increased excitability revealed here with 4AP. Similarly, the shift from hyperpolarizing towards depolarizing actions (Lagostena et al., 2010) has been proposed to aggravate increased excitability resulting from loss of GABAergic neurons. Moreover, in the J20 line of hAPP_FAD_ mice, PV neurons displayed greater depolarization of the resting membrane potential and reduced action potential amplitude (Verret et al., 2012). However, other alterations, such as increased numbers of NPY-expressing cells and GABAergic sprouting in the DG, may counteract the AD-related hyperexcitability (Palop et al., 2007) through compensatory remodeling of GABAergic neuronal population composition.

Finally, our data show that the decrease in PV-IR and NPY-IR cells is present at 1 month, whereas the number of NeuN-IR cells is unaltered, suggesting that phenotypic alterations of GABAergic neurons occur without neuronal loss at this early AD pathogenic stage. Functional changes manifesting as decreased PV and NPY expression, and increased synaptic excitability in CA1, all occur well before GABAergic cell death, which is not detectable before 6 months in TgCRND8 (Krantic et al., 2012; Albuquerque et al., 2015) and AβPPdE9 (Ramos et al., 2006) mice. This represents additional evidence supporting the hypothesis that hyperexcitability and increased seizure susceptibility are the cause rather than the consequence of AD-related neuronal death (Palop et al., 2007). Moreover, our study suggests that at early stages of AD-related pathology, GABAergic neurons downregulate at least some of their neurochemical markers (PV, NPY) but remain present, as no neuronal loss was detected by stereological quantification of NeuN-IR cells. A similar phenomenon has been observed in schizophrenia, both in animal models (Nullmeier et al., 2011) and patients (Akbarian et al., 1995; Impagnatiello et al., 1998; Guidotti et al., 2000; Hashimoto et al., 2003; Lewis et al., 2005).

In conclusion, this study reveals early alterations in hippocampal neuronal phenotypes that are associated with a functional increase in oscillatory activity and precede Aβ accumulation. These findings suggest that AD etiology may involve hippocampal GABAergic alterations occurring before appearance of symptoms and plaque formation.

## Author Contributions

IM, SM-R, SW, NM, RQ and SK designed experiments; IM, MSA, SM-R, CC, MAD and J-GC performed experiments; IM, MSA, SM-R, CC and SK analyzed data; IM, MSA, SM-R, CC, SW, NM and SK wrote the manuscript.

## Conflict of Interest Statement

The authors declare that the research was conducted in the absence of any commercial or financial relationships that could be construed as a potential conflict of interest.

## Abbreviations

Aβ: Amyloid beta
AICD: Amyloid precursor protein intracellular domain
βCTF: C-terminal fragment beta
AD: Alzheimer’s disease
CA: Cornu ammonis
DG: Dentate gyrus
GABA: γ-aminobutyric acid
GR: Granule cell layer
MO: Molecular layer
NPY: Neuropeptide Y
PO: Polymorphic layer
PV: Parvalbumin
PY: Pyramidal layer
SO: Stratum oriens
SR: Stratum radiatum.
